# μ-Adipato-κ^2^ *O* ^1^:*O* ^4^-bis­{[2,6-bis­(1*H*-benzimidazol-2-yl-κ*N* ^3^)pyridine-κ*N*](nitrato-κ*O*)lead(II)}

**DOI:** 10.1107/S1600536809052647

**Published:** 2009-12-12

**Authors:** Lian-Qiang Wei, Ming-Hua Zeng, Seik Weng Ng

**Affiliations:** a School of Chemistry & Chemical Engineering, Guangxi Normal University, 541004 Guilin 541004, People’s Republic of China; b Department of Chemistry, University of Malaya, 50603 Kuala Lumpur, Malaysia

## Abstract

The dinuclear title compound, [Pb_2_(C_6_H_8_O_4_)(NO_3_)_2_(C_19_H_13_N_5_)_2_], lies with the mid-point of the butyl chain of the bridging adipate unit on a center of inversion. The Pb^II^ ion is covalently bonded to the nitrate anion and is bonded to a carboxyl­ate group of the adipate unit by another covalent bond. The *N*-heterocycle functions in a chelating tridentate mode. The metal atom exists in a Ψ-octa­hedral coordination environment. When weaker Pb⋯O inter­actions are also considered, the geometry is a Ψ-tricapped trigonal prism in which the lone-pair electrons occupy one face of the trigonal prism. Adjacent mol­ecules are linked into a layer structure by N—H⋯O hydrogen bonds.

## Related literature

For the structure of a related Pb^II^ complex and its lone-pair sterechemistry, see: Meng *et al.* (2009 ▶).
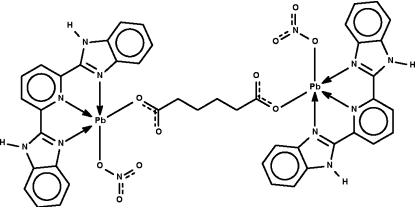


## Experimental

### 

#### Crystal data

[Pb_2_(C_6_H_8_O_4_)(NO_3_)_2_(C_19_H_13_N_5_)_2_]
*M* *_r_* = 1305.21
Triclinic, 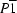

*a* = 9.3470 (7) Å
*b* = 10.6433 (8) Å
*c* = 11.3776 (8) Å
α = 106.696 (1)°
β = 95.343 (1)°
γ = 99.117 (1)°
*V* = 1058.9 (1) Å^3^
*Z* = 1
Mo *K*α radiation
μ = 8.02 mm^−1^
*T* = 293 K
0.26 × 0.12 × 0.08 mm

#### Data collection

Bruker APEXII diffractometer
Absorption correction: multi-scan (*SADABS*; Sheldrick, 1996 ▶) *T*_min_ = 0.230, *T*_max_ = 0.566
8299 measured reflections
4561 independent reflections
3586 reflections with *I* > 2σ(*I*)
*R*_int_ = 0.036

#### Refinement

*R*[*F* ^2^ > 2σ(*F* ^2^)] = 0.041
*wR*(*F* ^2^) = 0.099
*S* = 1.02
4561 reflections
307 parameters
H-atom parameters constrained
Δρ_max_ = 1.93 e Å^−3^
Δρ_min_ = −1.17 e Å^−3^

### 

Data collection: *APEX2* (Bruker, 2004 ▶); cell refinement: *SAINT* (Bruker, 2004 ▶); data reduction: *SAINT*; program(s) used to solve structure: *SHELXS97* (Sheldrick, 2008 ▶); program(s) used to refine structure: *SHELXL97* (Sheldrick, 2008 ▶); molecular graphics: *X-SEED* (Barbour, 2001 ▶); software used to prepare material for publication: *publCIF* (Westrip, 2009 ▶).

## Supplementary Material

Crystal structure: contains datablocks I, global. DOI: 10.1107/S1600536809052647/bt5131sup1.cif

Structure factors: contains datablocks I. DOI: 10.1107/S1600536809052647/bt5131Isup2.hkl

Additional supplementary materials:  crystallographic information; 3D view; checkCIF report

## Figures and Tables

**Table 1:** Hydrogen-bond geometry (Å, °)

*D*—H⋯*A*	*D*—H	H⋯*A*	*D*⋯*A*	*D*—H⋯*A*
N2—H2⋯O1^i^	0.86	1.95	2.744 (8)	152
N5—H5⋯O3^ii^	0.86	2.10	2.891 (9)	153

Symmetry codes: (i) 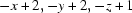; (ii) 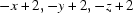.

**Fig. 1.**
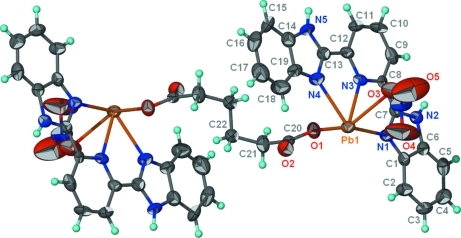
Anisotropic displacement ellipsoid plot (Barbour, 2001) of Pb2(NO3)2(C6H8O4)(C19H13N5)2 at the 70% probability level; hydrogen atoms are drawn as sphere of arbitrary radius.

**Fig. 2.**
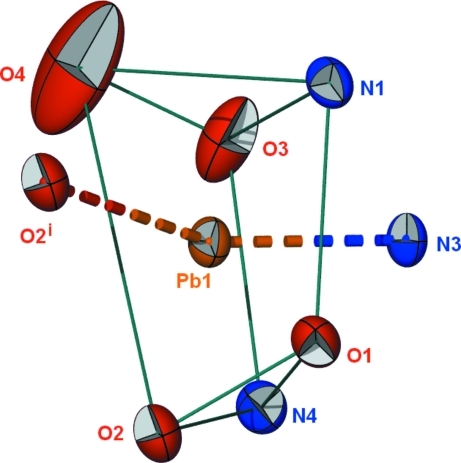
Detail of the coordination environment of the PbII centre.

## supplementary crystallographic information

### Experimental

Lead nitrate (0.250 mmol), 2-(6-(1*H*-benzo[*d*]imidazol-2-yl)pyridin-2-yl)-1*H*-benzo[*d*]imidazole (0.250 mmol), adipic acid (0.125 mmol) and water (10 ml) were sealed in a 25 ml Teflon-lined, stainless-steel Parr bomb. The bomb was heated at 413 K for 4 days and cooled to room temperature. Brown block-shaped crystals were colleacted and washed in water; the yield was 25%.

### Refinement

Hydrogen atoms were generated geometrically and were constrained to ride on their parent atoms [C–H = 0.93, N–H 0.86 Å; *U*_iso_(H) =1.2*U*_eq_(C,*N*)].

The final difference Fourier map had a peak near C14 and a hole near H2a.

### Crystal data

**Table:**

[Pb_2_(C_6_H_8_O_4_)(NO_3_)_2_(C_19_H_13_N_5_)_2_]	*Z* = 1
*M**_r_* = 1305.21	*F*(000) = 626
Triclinic, *P*1	*D*_x_ = 2.047 Mg m^−^^3^
Hall symbol: -P 1	Mo *K*α radiation, λ = 0.71073 Å
*a* = 9.3470 (7) Å	Cell parameters from 3150 reflections
*b* = 10.6433 (8) Å	θ = 2.2–24.8°
*c* = 11.3776 (8) Å	µ = 8.02 mm^−^^1^
α = 106.696 (1)°	*T* = 293 K
β = 95.343 (1)°	Block, brown
γ = 99.117 (1)°	0.26 × 0.12 × 0.08 mm
*V* = 1058.9 (1) Å^3^	

### Data collection

**Table:**

Bruker APEXII diffractometer	4561 independent reflections
Radiation source: fine-focus sealed tube	3586 reflections with *I* > 2σ(*I*)
graphite	*R*_int_ = 0.036
φ and ω scans	θ_max_ = 27.1°, θ_min_ = 1.9°
Absorption correction: multi-scan (*SADABS*; Sheldrick, 1996)	*h* = −11→11
*T*_min_ = 0.230, *T*_max_ = 0.566	*k* = −13→13
8299 measured reflections	*l* = −14→14

### Refinement

**Table:**

Refinement on *F*^2^	Primary atom site location: structure-invariant direct methods
Least-squares matrix: full	Secondary atom site location: difference Fourier map
*R*[*F*^2^ > 2σ(*F*^2^)] = 0.041	Hydrogen site location: inferred from neighbouring sites
*wR*(*F*^2^) = 0.099	H-atom parameters constrained
*S* = 1.02	*w* = 1/[σ^2^(*F*_o_^2^) + (0.0524*P*)^2^] where *P* = (*F*_o_^2^ + 2*F*_c_^2^)/3
4561 reflections	(Δ/σ)_max_ = 0.001
307 parameters	Δρ_max_ = 1.93 e Å^−^^3^
0 restraints	Δρ_min_ = −1.17 e Å^−^^3^

### Fractional atomic coordinates and isotropic or equivalent isotropic displacement parameters (Å^2^)

**Table:**

	*x*	*y*	*z*	*U*_iso_*/*U*_eq_	
Pb1	1.07669 (3)	0.71714 (3)	0.65491 (3)	0.02682 (11)	
O1	0.8637 (6)	0.6980 (5)	0.5092 (5)	0.0341 (13)	
O2	0.8304 (6)	0.4985 (5)	0.5330 (5)	0.0381 (13)	
O3	1.2560 (7)	0.8902 (7)	0.8623 (6)	0.0579 (18)	
O4	1.3952 (11)	0.7543 (10)	0.8022 (10)	0.126 (5)	
O5	1.4055 (12)	0.8554 (14)	0.9916 (9)	0.141 (5)	
N1	1.1854 (7)	0.8782 (6)	0.5449 (5)	0.0286 (14)	
N2	1.2263 (6)	1.0797 (6)	0.5237 (5)	0.0274 (14)	
H2	1.2215	1.1621	0.5343	0.033*	
N3	1.0091 (7)	0.9473 (6)	0.7236 (5)	0.0251 (13)	
N4	0.8962 (7)	0.7470 (6)	0.8149 (6)	0.0329 (15)	
N5	0.7760 (7)	0.8747 (6)	0.9451 (6)	0.0352 (16)	
H5	0.7435	0.9440	0.9839	0.042*	
N6	1.3504 (9)	0.8307 (8)	0.8859 (7)	0.052 (2)	
C1	1.2754 (8)	0.8763 (7)	0.4564 (7)	0.0262 (16)	
C2	1.3328 (9)	0.7741 (8)	0.3826 (7)	0.0324 (18)	
H2A	1.3132	0.6882	0.3893	0.039*	
C3	1.4197 (9)	0.8036 (8)	0.2989 (7)	0.0349 (19)	
H3	1.4600	0.7366	0.2490	0.042*	
C4	1.4490 (9)	0.9331 (9)	0.2873 (7)	0.0369 (19)	
H4	1.5085	0.9499	0.2303	0.044*	
C5	1.3918 (8)	1.0343 (8)	0.3581 (7)	0.0350 (19)	
H5A	1.4106	1.1200	0.3508	0.042*	
C6	1.3046 (8)	1.0032 (7)	0.4412 (7)	0.0278 (16)	
C7	1.1608 (8)	1.0009 (7)	0.5828 (6)	0.0258 (16)	
C8	1.0701 (7)	1.0443 (7)	0.6764 (6)	0.0208 (14)	
C9	1.0481 (8)	1.1737 (7)	0.7213 (8)	0.0344 (18)	
H9	1.0887	1.2392	0.6882	0.041*	
C10	0.9640 (9)	1.2052 (8)	0.8172 (7)	0.038 (2)	
H10	0.9509	1.2926	0.8495	0.045*	
C11	0.9004 (9)	1.1077 (8)	0.8642 (7)	0.0340 (18)	
H11	0.8428	1.1276	0.9269	0.041*	
C12	0.9248 (8)	0.9783 (7)	0.8148 (7)	0.0263 (16)	
C13	0.8642 (8)	0.8677 (8)	0.8563 (7)	0.0299 (17)	
C14	0.7477 (9)	0.7502 (8)	0.9616 (8)	0.0345 (18)	
C15	0.6696 (9)	0.7026 (9)	1.0431 (8)	0.041 (2)	
H15	0.6169	0.7550	1.0960	0.049*	
C16	0.6744 (9)	0.5751 (10)	1.0410 (8)	0.046 (2)	
H16	0.6258	0.5401	1.0958	0.056*	
C17	0.7503 (11)	0.4942 (10)	0.9588 (9)	0.056 (3)	
H17	0.7483	0.4066	0.9590	0.067*	
C18	0.8266 (11)	0.5409 (9)	0.8793 (9)	0.050 (2)	
H18	0.8782	0.4876	0.8261	0.060*	
C19	0.8245 (9)	0.6720 (8)	0.8805 (7)	0.0333 (18)	
C20	0.7885 (8)	0.5807 (7)	0.4878 (7)	0.0264 (16)	
C21	0.6450 (8)	0.5467 (8)	0.4015 (8)	0.039 (2)	
H21A	0.6606	0.4989	0.3187	0.047*	
H21B	0.6159	0.6292	0.3982	0.047*	
C22	0.5208 (9)	0.4628 (8)	0.4384 (7)	0.038 (2)	
H22A	0.5500	0.3811	0.4443	0.045*	
H22B	0.4361	0.4383	0.3746	0.045*	

### Atomic displacement parameters (Å^2^)

**Table:**

	*U*^11^	*U*^22^	*U*^33^	*U*^12^	*U*^13^	*U*^23^
Pb1	0.02973 (17)	0.02289 (16)	0.03042 (16)	0.00981 (11)	0.00830 (11)	0.00846 (11)
O1	0.030 (3)	0.025 (3)	0.046 (3)	0.003 (2)	0.003 (3)	0.013 (2)
O2	0.041 (3)	0.027 (3)	0.051 (4)	0.009 (3)	0.010 (3)	0.016 (3)
O3	0.053 (4)	0.060 (4)	0.052 (4)	0.031 (4)	−0.009 (3)	−0.001 (3)
O4	0.127 (9)	0.126 (9)	0.097 (7)	0.085 (7)	−0.009 (6)	−0.034 (6)
O5	0.122 (9)	0.244 (14)	0.055 (6)	0.098 (9)	−0.008 (6)	0.017 (7)
N1	0.035 (4)	0.022 (3)	0.030 (3)	0.007 (3)	0.011 (3)	0.008 (3)
N2	0.030 (3)	0.022 (3)	0.031 (3)	0.006 (3)	0.007 (3)	0.008 (3)
N3	0.031 (3)	0.019 (3)	0.025 (3)	0.009 (3)	0.003 (3)	0.004 (2)
N4	0.038 (4)	0.029 (4)	0.036 (4)	0.011 (3)	0.016 (3)	0.010 (3)
N5	0.033 (4)	0.033 (4)	0.039 (4)	0.010 (3)	0.019 (3)	0.004 (3)
N6	0.057 (5)	0.062 (5)	0.037 (4)	0.029 (4)	0.006 (4)	0.005 (4)
C1	0.022 (4)	0.025 (4)	0.028 (4)	0.002 (3)	−0.002 (3)	0.007 (3)
C2	0.042 (5)	0.027 (4)	0.032 (4)	0.014 (4)	0.011 (4)	0.010 (3)
C3	0.036 (5)	0.042 (5)	0.027 (4)	0.014 (4)	0.010 (3)	0.006 (3)
C4	0.036 (5)	0.050 (5)	0.027 (4)	0.007 (4)	0.007 (4)	0.015 (4)
C5	0.033 (4)	0.033 (4)	0.040 (5)	−0.003 (4)	0.002 (4)	0.019 (4)
C6	0.022 (4)	0.030 (4)	0.028 (4)	0.004 (3)	0.000 (3)	0.005 (3)
C7	0.025 (4)	0.024 (4)	0.024 (4)	0.000 (3)	0.001 (3)	0.004 (3)
C8	0.009 (3)	0.027 (4)	0.020 (3)	0.002 (3)	−0.007 (3)	0.002 (3)
C9	0.032 (4)	0.022 (4)	0.045 (5)	0.000 (3)	−0.005 (4)	0.012 (3)
C10	0.050 (5)	0.023 (4)	0.039 (5)	0.018 (4)	0.011 (4)	−0.001 (3)
C11	0.034 (4)	0.034 (4)	0.031 (4)	0.009 (4)	0.013 (3)	0.001 (3)
C12	0.029 (4)	0.019 (4)	0.030 (4)	0.010 (3)	0.003 (3)	0.003 (3)
C13	0.028 (4)	0.029 (4)	0.029 (4)	0.005 (3)	0.002 (3)	0.005 (3)
C14	0.034 (5)	0.027 (4)	0.042 (5)	0.004 (3)	0.005 (4)	0.011 (4)
C15	0.027 (4)	0.049 (5)	0.048 (5)	0.003 (4)	0.018 (4)	0.015 (4)
C16	0.038 (5)	0.060 (6)	0.047 (5)	−0.003 (4)	0.012 (4)	0.029 (5)
C17	0.068 (7)	0.047 (6)	0.046 (6)	−0.006 (5)	0.008 (5)	0.013 (5)
C18	0.070 (7)	0.032 (5)	0.054 (6)	0.016 (5)	0.027 (5)	0.014 (4)
C19	0.033 (4)	0.032 (4)	0.035 (4)	0.009 (4)	0.012 (4)	0.007 (3)
C20	0.026 (4)	0.019 (4)	0.031 (4)	0.003 (3)	0.012 (3)	0.002 (3)
C21	0.036 (5)	0.036 (5)	0.041 (5)	−0.005 (4)	0.004 (4)	0.012 (4)
C22	0.032 (4)	0.035 (5)	0.036 (5)	0.000 (4)	0.006 (4)	−0.001 (4)

### Geometric parameters (Å, °)

**Table:**

Pb1—O1	2.411 (5)	C4—C5	1.364 (11)
Pb1—N1	2.541 (6)	C4—H4	0.9300
Pb1—N3	2.548 (6)	C5—C6	1.380 (11)
Pb1—N4	2.583 (6)	C5—H5A	0.9300
Pb1—O3	2.749 (6)	C7—C8	1.441 (10)
Pb1—O2	2.914 (6)	C8—C9	1.381 (10)
Pb1—O2^i^	2.958 (5)	C9—C10	1.397 (11)
Pb1—O4	3.185 (10)	C9—H9	0.9300
O1—C20	1.275 (9)	C10—C11	1.378 (11)
O2—C20	1.231 (9)	C10—H10	0.9300
O3—N6	1.217 (9)	C11—C12	1.393 (10)
O4—N6	1.216 (11)	C11—H11	0.9300
O5—N6	1.200 (11)	C12—C13	1.445 (11)
N1—C7	1.317 (9)	C14—C15	1.389 (11)
N1—C1	1.369 (9)	C14—C19	1.389 (11)
N2—C7	1.330 (9)	C15—C16	1.359 (12)
N2—C6	1.399 (9)	C15—H15	0.9300
N2—H2	0.8600	C16—C17	1.405 (13)
N3—C12	1.358 (9)	C16—H16	0.9300
N3—C8	1.367 (9)	C17—C18	1.356 (13)
N4—C13	1.327 (9)	C17—H17	0.9300
N4—C19	1.382 (10)	C18—C19	1.394 (12)
N5—C13	1.356 (9)	C18—H18	0.9300
N5—C14	1.379 (10)	C20—C21	1.513 (11)
N5—H5	0.8600	C21—C22	1.516 (11)
C1—C2	1.386 (10)	C21—H21A	0.9700
C1—C6	1.399 (10)	C21—H21B	0.9700
C2—C3	1.378 (11)	C22—C22^ii^	1.519 (14)
C2—H2A	0.9300	C22—H22A	0.9700
C3—C4	1.408 (11)	C22—H22B	0.9700
C3—H3	0.9300		
			
O1—Pb1—N1	81.15 (19)	C3—C4—H4	119.3
O1—Pb1—N3	76.12 (18)	C4—C5—C6	116.6 (7)
N1—Pb1—N3	65.32 (19)	C4—C5—H5A	121.7
O1—Pb1—N4	83.9 (2)	C6—C5—H5A	121.7
N1—Pb1—N4	129.70 (19)	C5—C6—C1	123.5 (7)
N3—Pb1—N4	64.51 (19)	C5—C6—N2	131.7 (7)
O1—Pb1—O3	142.75 (18)	C1—C6—N2	104.8 (6)
N1—Pb1—O3	84.8 (2)	N1—C7—N2	112.8 (6)
N3—Pb1—O3	66.66 (19)	N1—C7—C8	123.6 (7)
N4—Pb1—O3	78.9 (2)	N2—C7—C8	123.6 (7)
O1—Pb1—O2	47.51 (16)	N3—C8—C9	120.6 (6)
N1—Pb1—O2	121.65 (18)	N3—C8—C7	114.8 (6)
N3—Pb1—O2	114.54 (17)	C9—C8—C7	124.5 (7)
N4—Pb1—O2	77.49 (18)	C8—C9—C10	119.2 (7)
O3—Pb1—O2	152.3 (2)	C8—C9—H9	120.4
O1—Pb1—O2^i^	89.14 (17)	C10—C9—H9	120.4
N1—Pb1—O2^i^	87.79 (17)	C11—C10—C9	120.6 (7)
N3—Pb1—O2^i^	150.76 (17)	C11—C10—H10	119.7
N4—Pb1—O2^i^	139.77 (18)	C9—C10—H10	119.7
O3—Pb1—O2^i^	124.72 (17)	C10—C11—C12	118.0 (7)
O2—Pb1—O2^i^	68.94 (18)	C10—C11—H11	121.0
O1—Pb1—O4	167.8 (3)	C12—C11—H11	121.0
N1—Pb1—O4	88.3 (3)	N3—C12—C11	121.9 (7)
N3—Pb1—O4	105.0 (2)	N3—C12—C13	114.9 (6)
N4—Pb1—O4	107.6 (2)	C11—C12—C13	123.2 (7)
O3—Pb1—O4	40.80 (19)	N4—C13—N5	111.9 (7)
O2—Pb1—O4	137.4 (2)	N4—C13—C12	122.8 (7)
O2^i^—Pb1—O4	84.36 (19)	N5—C13—C12	125.2 (7)
C20—O1—Pb1	106.6 (5)	N5—C14—C15	131.9 (8)
C20—O2—Pb1	83.5 (4)	N5—C14—C19	105.6 (7)
N6—O3—Pb1	105.7 (5)	C15—C14—C19	122.4 (8)
N6—O4—Pb1	84.3 (6)	C16—C15—C14	116.2 (8)
C7—N1—C1	106.2 (6)	C16—C15—H15	121.9
C7—N1—Pb1	116.8 (4)	C14—C15—H15	121.9
C1—N1—Pb1	136.9 (5)	C15—C16—C17	122.1 (8)
C7—N2—C6	107.1 (6)	C15—C16—H16	118.9
C7—N2—H2	126.5	C17—C16—H16	118.9
C6—N2—H2	126.5	C18—C17—C16	121.6 (9)
C12—N3—C8	119.6 (6)	C18—C17—H17	119.2
C12—N3—Pb1	120.8 (5)	C16—C17—H17	119.2
C8—N3—Pb1	119.3 (4)	C17—C18—C19	117.3 (9)
C13—N4—C19	105.6 (6)	C17—C18—H18	121.3
C13—N4—Pb1	116.7 (5)	C19—C18—H18	121.3
C19—N4—Pb1	137.6 (5)	N4—C19—C14	109.5 (7)
C13—N5—C14	107.4 (6)	N4—C19—C18	130.0 (7)
C13—N5—H5	126.3	C14—C19—C18	120.4 (8)
C14—N5—H5	126.3	O2—C20—O1	122.3 (7)
O5—N6—O4	120.5 (10)	O2—C20—C21	121.4 (7)
O5—N6—O3	119.2 (9)	O1—C20—C21	116.4 (7)
O4—N6—O3	120.1 (9)	C20—C21—C22	114.5 (7)
N1—C1—C2	131.7 (7)	C20—C21—H21A	108.6
N1—C1—C6	109.1 (6)	C22—C21—H21A	108.6
C2—C1—C6	119.2 (7)	C20—C21—H21B	108.6
C3—C2—C1	118.0 (8)	C22—C21—H21B	108.6
C3—C2—H2A	121.0	H21A—C21—H21B	107.6
C1—C2—H2A	121.0	C21—C22—C22^ii^	111.7 (8)
C2—C3—C4	121.4 (7)	C21—C22—H22A	109.3
C2—C3—H3	119.3	C22^ii^—C22—H22A	109.3
C4—C3—H3	119.3	C21—C22—H22B	109.3
C5—C4—C3	121.3 (7)	C22^ii^—C22—H22B	109.3
C5—C4—H4	119.3	H22A—C22—H22B	107.9
			
N1—Pb1—O1—C20	151.2 (5)	Pb1—N1—C1—C2	7.0 (13)
N3—Pb1—O1—C20	−142.2 (5)	C7—N1—C1—C6	−0.2 (8)
N4—Pb1—O1—C20	−77.0 (5)	Pb1—N1—C1—C6	−175.5 (5)
O3—Pb1—O1—C20	−139.6 (5)	N1—C1—C2—C3	178.9 (8)
O2—Pb1—O1—C20	1.6 (4)	C6—C1—C2—C3	1.6 (11)
O2^i^—Pb1—O1—C20	63.3 (5)	C1—C2—C3—C4	−0.5 (12)
O4—Pb1—O1—C20	120.9 (10)	C2—C3—C4—C5	−0.3 (13)
O1—Pb1—O2—C20	−1.6 (4)	C3—C4—C5—C6	0.1 (12)
N1—Pb1—O2—C20	−37.5 (5)	C4—C5—C6—C1	1.0 (12)
N3—Pb1—O2—C20	37.6 (5)	C4—C5—C6—N2	−177.8 (8)
N4—Pb1—O2—C20	91.7 (4)	N1—C1—C6—C5	−179.8 (7)
O3—Pb1—O2—C20	123.7 (5)	C2—C1—C6—C5	−1.9 (12)
O2^i^—Pb1—O2—C20	−110.9 (5)	N1—C1—C6—N2	−0.7 (8)
O4—Pb1—O2—C20	−165.8 (4)	C2—C1—C6—N2	177.2 (7)
O1—Pb1—O3—N6	−178.2 (5)	C7—N2—C6—C5	−179.7 (8)
N1—Pb1—O3—N6	−110.1 (6)	C7—N2—C6—C1	1.3 (8)
N3—Pb1—O3—N6	−175.4 (7)	C1—N1—C7—N2	1.1 (9)
N4—Pb1—O3—N6	117.7 (6)	Pb1—N1—C7—N2	177.5 (5)
O2—Pb1—O3—N6	85.9 (7)	C1—N1—C7—C8	179.9 (7)
O2^i^—Pb1—O3—N6	−26.4 (7)	Pb1—N1—C7—C8	−3.7 (9)
O4—Pb1—O3—N6	−16.7 (6)	C6—N2—C7—N1	−1.6 (9)
O1—Pb1—O4—N6	130.1 (9)	C6—N2—C7—C8	179.7 (6)
N1—Pb1—O4—N6	100.2 (8)	C12—N3—C8—C9	0.7 (10)
N3—Pb1—O4—N6	36.4 (8)	Pb1—N3—C8—C9	−173.1 (5)
N4—Pb1—O4—N6	−31.1 (8)	C12—N3—C8—C7	178.2 (6)
O3—Pb1—O4—N6	16.2 (6)	Pb1—N3—C8—C7	4.4 (8)
O2—Pb1—O4—N6	−121.7 (7)	N1—C7—C8—N3	−0.4 (10)
O2^i^—Pb1—O4—N6	−171.8 (8)	N2—C7—C8—N3	178.2 (6)
O1—Pb1—N1—C7	82.8 (5)	N1—C7—C8—C9	177.0 (7)
N3—Pb1—N1—C7	4.0 (5)	N2—C7—C8—C9	−4.4 (11)
N4—Pb1—N1—C7	8.3 (6)	N3—C8—C9—C10	0.8 (11)
O3—Pb1—N1—C7	−62.6 (5)	C7—C8—C9—C10	−176.4 (7)
O2—Pb1—N1—C7	108.7 (5)	C8—C9—C10—C11	−1.8 (12)
O2^i^—Pb1—N1—C7	172.2 (5)	C9—C10—C11—C12	1.2 (12)
O4—Pb1—N1—C7	−103.4 (5)	C8—N3—C12—C11	−1.3 (11)
O1—Pb1—N1—C1	−102.3 (7)	Pb1—N3—C12—C11	172.4 (6)
N3—Pb1—N1—C1	179.0 (8)	C8—N3—C12—C13	179.1 (6)
N4—Pb1—N1—C1	−176.8 (6)	Pb1—N3—C12—C13	−7.3 (9)
O3—Pb1—N1—C1	112.3 (7)	C10—C11—C12—N3	0.4 (12)
O2—Pb1—N1—C1	−76.3 (7)	C10—C11—C12—C13	180.0 (8)
O2^i^—Pb1—N1—C1	−12.8 (7)	C19—N4—C13—N5	0.0 (9)
O4—Pb1—N1—C1	71.6 (7)	Pb1—N4—C13—N5	−176.3 (5)
O1—Pb1—N3—C12	95.3 (6)	C19—N4—C13—C12	176.8 (7)
N1—Pb1—N3—C12	−178.2 (6)	Pb1—N4—C13—C12	0.5 (10)
N4—Pb1—N3—C12	5.5 (5)	C14—N5—C13—N4	−0.6 (9)
O3—Pb1—N3—C12	−83.0 (6)	C14—N5—C13—C12	−177.2 (7)
O2—Pb1—N3—C12	66.7 (6)	N3—C12—C13—N4	4.4 (11)
O2^i^—Pb1—N3—C12	157.1 (5)	C11—C12—C13—N4	−175.2 (8)
O4—Pb1—N3—C12	−97.2 (6)	N3—C12—C13—N5	−179.3 (7)
O1—Pb1—N3—C8	−91.0 (5)	C11—C12—C13—N5	1.1 (12)
N1—Pb1—N3—C8	−4.5 (5)	C13—N5—C14—C15	177.1 (9)
N4—Pb1—N3—C8	179.2 (5)	C13—N5—C14—C19	0.8 (9)
O3—Pb1—N3—C8	90.7 (5)	N5—C14—C15—C16	−174.5 (9)
O2—Pb1—N3—C8	−119.6 (5)	C19—C14—C15—C16	1.2 (13)
O2^i^—Pb1—N3—C8	−29.2 (7)	C14—C15—C16—C17	−1.8 (14)
O4—Pb1—N3—C8	76.5 (5)	C15—C16—C17—C18	1.9 (16)
O1—Pb1—N4—C13	−80.3 (6)	C16—C17—C18—C19	−1.2 (16)
N1—Pb1—N4—C13	−7.2 (7)	C13—N4—C19—C14	0.5 (9)
N3—Pb1—N4—C13	−2.9 (5)	Pb1—N4—C19—C14	175.6 (6)
O3—Pb1—N4—C13	66.4 (6)	C13—N4—C19—C18	−176.0 (9)
O2—Pb1—N4—C13	−128.1 (6)	Pb1—N4—C19—C18	−0.9 (15)
O2^i^—Pb1—N4—C13	−161.8 (5)	N5—C14—C19—N4	−0.8 (9)
O4—Pb1—N4—C13	95.7 (6)	C15—C14—C19—N4	−177.5 (8)
O1—Pb1—N4—C19	104.9 (8)	N5—C14—C19—C18	176.1 (8)
N1—Pb1—N4—C19	178.1 (7)	C15—C14—C19—C18	−0.6 (13)
N3—Pb1—N4—C19	−177.6 (9)	C17—C18—C19—N4	176.8 (9)
O3—Pb1—N4—C19	−108.3 (8)	C17—C18—C19—C14	0.6 (14)
O2—Pb1—N4—C19	57.2 (8)	Pb1—O2—C20—O1	2.6 (7)
O2^i^—Pb1—N4—C19	23.5 (9)	Pb1—O2—C20—C21	−178.5 (7)
O4—Pb1—N4—C19	−79.0 (8)	Pb1—O1—C20—O2	−3.2 (8)
Pb1—O4—N6—O5	156.9 (12)	Pb1—O1—C20—C21	177.8 (5)
Pb1—O4—N6—O3	−28.4 (10)	O2—C20—C21—C22	39.6 (10)
Pb1—O3—N6—O5	−150.5 (10)	O1—C20—C21—C22	−141.5 (7)
Pb1—O3—N6—O4	34.7 (12)	C20—C21—C22—C22^ii^	64.3 (12)
C7—N1—C1—C2	−177.7 (8)		

Symmetry codes: (i) −*x*+2, −*y*+1, −*z*+1; (ii) −*x*+1, −*y*+1, −*z*+1.

### Hydrogen-bond geometry (Å, °)

**Table:**

*D*—H···*A*	*D*—H	H···*A*	*D*···*A*	*D*—H···*A*
N2—H2···O1^iii^	0.86	1.95	2.744 (8)	152
N5—H5···O3^iv^	0.86	2.10	2.891 (9)	153

Symmetry codes: (iii) −*x*+2, −*y*+2, −*z*+1; (iv) −*x*+2, −*y*+2, −*z*+2.
